# Effects of a maternal ketogenic diet on maternal and offspring metabolic health in mice

**DOI:** 10.1371/journal.pone.0356015

**Published:** 2026-10-21

**Authors:** Antonio H. Ruano, Rachel A. Kracaw, Isabella A. Cervantes, Simone Hernandez Ruano, Moises J. Tacam, Kathleen A. Pennington

**Affiliations:** Department of Obstetrics and Gynecology, Baylor College of Medicine, Houston, TX, United States of America; Universidad de Murcia, SPAIN

## Abstract

Ketogenic (keto) diets have gained popularity due to their potential benefits on weight loss and metabolic conditions. However, the consequences of consuming a keto diet before, during, and after pregnancy on maternal and offspring health remain incompletely understood. To investigate this, female Swiss Webster mice were randomly assigned to either a keto or control diet. After 6 weeks on diet, females were mated and maintained on their respective diets throughout pregnancy and lactation. In experiment 1, glucose tolerance tests (GTT) were performed on pregnant dams at gestational day (GD) 16.5. At GD 17.5, dams were euthanized, urine was collected for ketone analysis, and fetal and placental weights were recorded. In experiment 2, dams delivered naturally and one male and one female offspring per dam were retained and weighed weekly. At 12 weeks of age, offspring underwent GTT, followed by euthanasia and serum collection for insulin and leptin analysis. Compared with controls, keto dams exhibited increased body weight, impaired glucose tolerance, elevated urinary ketones, and reduced circulating insulin during late gestation (p < 0.05). Fetuses from keto dams were significantly smaller (p < 0.05), whereas placental weight and placental efficiency were unchanged. Offspring from keto dams tended to have lower body weights during early postnatal development; however, longitudinal analysis did not identify a significant overall effect of maternal diet or a maternal diet x time interaction. At 12 weeks, keto offspring exhibited a modest but statistically significant improvement in glucose tolerance compared to control offspring (p < 0.05). Male keto offspring had significantly decreased serum insulin (p < 0.05) compared to male control offspring, while no differences were observed in females. Serum leptin levels did not differ between diet groups, although sex differences present in control offspring were not observed in keto offspring. In this exploratory mouse study, ketogenic diet consumption was associated with altered maternal glucose homeostasis during pregnancy and reduced fetal growth, although these maternal and fetal findings were derived from a limited number of independent pregnancies and require confirmation in larger studies. Offspring exposed to maternal ketogenic diet demonstrated modest metabolic differences at 12 weeks of age. Together, these findings support further investigation of the maternal and developmental consequences of ketogenic diet exposure during pregnancy.

## Introduction

Obesity is a growing global epidemic that contributes to increased health risks and significant economic burden [1]. Obese individuals are at heightened risk for metabolic syndrome, a condition characterized by central obesity, diabetes, hypertension, and dyslipidemia [2]. In the United States, metabolic syndrome accounts for 6–7% of all mortalities [1,3,4]. Exposure to an adverse maternal environment during development is a well-established risk factor for later-life metabolic syndrome [2,3,5]. This observation was central to the formulation of the ‘developmental origins of health and disease’ (DOHaD) hypothesis, also referred to as fetal programming [3,6]. Maternal nutrition plays a key role in shaping the intrauterine environment, and numerous epidemiological studies in humans as well as animal models have demonstrated that poor maternal nutrition increases the risk of offspring developing metabolic syndrome, which includes non-alcoholic fatty liver disease (NAFLD), and cardiovascular disease (CVD), later in life [2,3,5].

Ketogenic (keto) diets, diets high in fat, moderate in protein and low in carbohydrates, have gained popularity for their potential benefits in weight loss, metabolic disorders, cardiovascular health, and neurological conditions [7–12]. However, adverse outcomes have also been reported, including increased cholesterol and triglycerides, as well as altered liver lipid metabolism, which are linked to diabetes, CVD, and hypertension [13–15]. More recently, keto diets have been recommended for women with polycystic ovary syndrome (PCOS), a condition affecting 8–13% of reproductive age women [14,16–18]. Despite the increasing number of reproductive aged women consuming a keto diet, the effects of a keto diet during pregnancy and on long-term offspring health remain incompletely understood, with only a limited number of animal and human studies addressing maternal and offspring outcomes [19–23].

Although keto diets have demonstrated metabolic benefits in non-pregnant populations [8,11,14,16], pregnancy represents a unique physiological state characterized by progressive insulin resistance, altered maternal nutrient partitioning, and increased metabolic demands to support fetal growth [24]. Consequently, maternal responses to keto diet consumption during pregnancy may differ substantially from those observed outside of pregnancy, highlighting the need for pregnancy-specific investigation. Animal models provide a valuable opportunity to examine maternal and fetal responses to keto diet exposure under controlled experimental conditions that cannot be readily achieved in humans. However, species-specific differences in energy metabolism and responses to keto diets should be considered when interpreting these findings and translating them to human pregnancy.

Animal studies provide early concerns: in rats gestational keto diet consumption impaired offspring somatic and neurological development [20], while in mice it altered neonatal brain structure and slowed physiologic growth [21,22]. These alterations in brain structure we subsequently linked to altered offspring behavior [23,25]. A recent study by Zala et al., demonstrated a ketogenic diet fed during gestation resulted in reduced litter size, altered sex ratio and had sex specific effects on the offspring [19]. However, to date, no studies have examined the impact of maternal keto diet on long-term offspring metabolic health.

The objective of this study was to evaluate the effects of keto diet on maternal and offspring metabolic health. We hypothesized that consumption of a low-carbohydrate, high-fat keto diet before, during and after pregnancy would alter maternal metabolic adaptations during pregnancy and influence offspring metabolic health. To test this, we evaluated the effects of maternal ketogenic diet consumption on maternal glucose homeostasis during late gestation, fetal growth, and offspring metabolic health using a mouse model.

## Materials and methods

### Animals

The authors assert that all procedures contributing to this work comply with the ethical standards of the relevant national guides on the care and use of laboratory animals (National Institute of Health (NIH) and were approved by the Baylor College of Medicine Institutional Animal Care and Use Committee (IACUC protocol #AN-7439). This study is reported in accordance with ARRIVE guidelines. Fig 1 is a schematic of the animal experimental design which is also described below.

**Fig 1:**
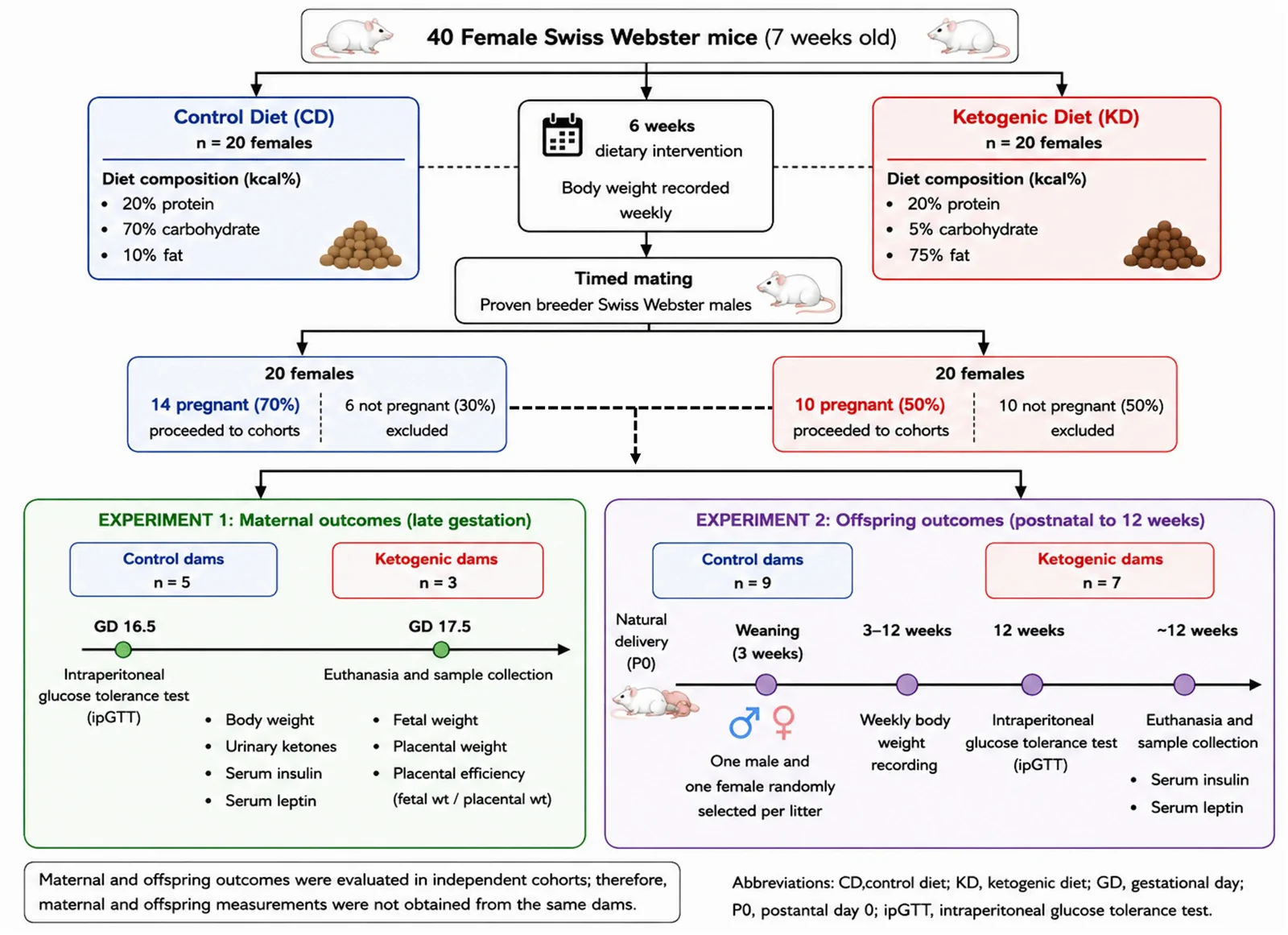
Experimental design and animal allocation for maternal and offspring studies. Forty female Swiss Webster mice (7 weeks of age) were assigned to either a control diet (CD; 20% protein, 70% carbohydrate, 10% fat; *n* = 20) or a ketogenic diet (KD; 20% protein, 5% carbohydrate, 75% fat; *n* = 20) for 6 weeks prior to timed mating with proven breeder males. Pregnancy rates were 70% (14/20) in the CD group and 50% (10/20) in the KD group. Pregnant females were allocated to one of two independent experimental cohorts. **Experiment 1** evaluated maternal metabolic adaptations during late gestation (CD: *n* = 5 dams; KD: *n* = 3 dams). An intraperitoneal glucose tolerance test (ipGTT) was performed on gestational day (GD) 16.5, followed by euthanasia and sample collection on GD17.5 for assessment of maternal body weight, urinary ketones, serum insulin, serum leptin, fetal weight, placental weight, and placental efficiency (fetal weight/placental weight). **Experiment 2** evaluated offspring outcomes from birth through 12 weeks of age (CD: *n* = 9 dams; KD: *n* = 7 dams). Following natural delivery, one male and one female offspring were randomly selected from each litter for longitudinal assessment. Offspring body weight was recorded weekly from 3–12 weeks of age. At 12 weeks, offspring underwent an ipGTT followed by euthanasia and collection of serum for insulin and leptin analyses. Maternal and offspring outcomes were evaluated in **independent cohorts**; therefore, maternal and offspring measurements were not obtained from the same dams. **Abbreviations:** CD, control diet; KD, ketogenic diet; GD, gestational day; P0, postnatal day 0; ipGTT, intraperitoneal glucose tolerance test.

Six-week-old female Swiss Webster mice were obtained from Charles Rivers Laboratories (Wilmington, Massachusetts, USA) and housed in a 14/10 hour light/dark cycle at 22–24°C with 40–60% humidity. At seven weeks of age, females, weighing 31.5 ± 0.3 grams (see supplementary data files), were randomly divided into two diet groups: control (n = 20; 20% protein, 70% carbohydrate, 10% fat; cat#: D12450K, Research Diets, New Brunswick, NJ, USA) or keto (n = 20; 20% protein, 5% carbohydrate, 75% fat; cat# D14090501 Research Diets) as previously described [26]. Detailed diet composition, including macronutrient sources and energy density, is provided in Supporting Information S1 Table. Following 6 weeks of diet exposure, females (approximately 13 weeks of age) were mated to proven breeder males (aged 15–20 weeks) in a ratio of 2 females to 1 male, and the day of copulatory plug was designated as gestational day (GD) 0.5. Following plug detection dams were maintained on their respective diets throughout pregnancy and lactation. Mice were provided ad libitum food and water as well as access to nesting materials. Reproductive characteristics can be found in Supporting Information S2 Table.

Because conception rates were lower than anticipated, particularly in the keto diet females, animals were allocated into two independent experimental cohorts to permit completion of both maternal and offspring outcomes. Experiment 1 was designed to evaluate maternal metabolic adaptations during late gestation with n = 5, and n = 3 keto dams. Experiment 2 was designed to evaluate long-term offspring outcomes with n = 9 control and n = 7 keto, for a conception rate of 70% for the control group and 50% in the keto group which was not significantly different between groups. Importantly, these cohorts were independent, and maternal and offspring outcomes were not derived from the same dams, therefore, correlation analysis between maternal and offspring phenotypes were not possible.

Experiment 1: At gestational day (GD) 16.5 glucose tolerance tests (GTT) were performed in control (n = 5) and keto (n = 3) dams, at GD 17.5 urine was collected by scruffing the dams and applying gentle pressure to the lower abdomen to stimulate urination. Urine was collected into a microcentrifuge tube and then immediately analyzed for urine ketones as described below. Dams were then euthanized via CO^2^ inhalation (30% chamber volume/min) followed immediately by cardiac puncture for blood collection. Blood was collected for future serum analysis and fetal and placental weights were obtained.

Experiment 2: Dams were allowed to deliver naturally. Litter size at birth was recorded; however litters were not standardized or culled. To minimize stress and the risk of pup cannibalism, litters were not disturbed immediately following birth. Consequently, individual pup body weights, maternal body weights during lactation, litter level sex ratios and neonatal survival were not recorded. At weaning, one male and one female offspring from each litter were retained for longitudinal analysis and weighed weekly until 12 weeks of age. In total 9 control and 7 keto dams gave birth, the other females (n = 6 control and n = 10 keto) were not pregnant and were euthanized. At 12 weeks of age, GTTs were performed. Offspring were euthanized at 12 weeks of age via CO^2^ inhalation (30% chamber volume/min) followed immediately by cardiac puncture for blood collection. Blood was collected for serum insulin and leptin analysis. For all studies, experimenters were blinded to animal treatment.

### Urine ketone analysis

Just prior to euthanasia urine was collected and then immediately applied to ketone test strips (PrimeScreen, Wondofo Biotech, Science City, Huangpu, Guangzhou, China). A visual reading was made and recorded.

### Intraperitoneal glucose tolerance tests

IPGTTs were performed in control and keto dams at GD 16.5 and 12 week old offspring as previously described [27–30] and according to the National Mouse Metabolic Phenotyping Centers protocols for IPGTT (https://www.mmpc.org/shared/document.aspx?id=141&docType=Protocol). Briefly, dams were fasted for 4 hours or offspring were fasted for 6 hours, and then a baseline fasting blood glucose sample was obtained from a venous tail sample. An intraperitoneal injection of glucose (2g/kg) was given. Blood glucose levels were obtained at 15-, 30-, 60- and 120-minutes post glucose injection by removing the tail scab for blood collection. All blood glucose measurements were performed in duplicate using two ReliOn Prime Blood Glucose Monitoring System meters (Walmart, Bentonville, AR, USA).

### Serum analysis

Serum insulin and leptin were measured in control and keto dams at GD 17.5 and in 12 week old control and keto offspring using a Rat/Mouse Insulin ELISA (EMD Millipore, Billerica, MA, USA) and Mouse Leptin ELISA (EMD Millipore), respectively, as previously described [29,30] and according to manufacturer’s instructions.

### Statistical analysis

Statistical analysis was performed using GraphPad Prism (La Jolla, CA, USA). For maternal ipGTT, data were analyzed using a two-way repeated-measures ANOVA with treatment and time as factors. Area under the curve (AUC) for ipGTT was calculated from the glucose concentration-time curve using the trapezoidal method and is expressed as mg·min/dL. For maternal data, AUC, urine keto levels, litter size, fetal and placental weights, serum insulin and leptin were analyzed using unpaired Student’s t-tests. Fetal and placental weights as well as placental efficiency were averaged within each litter, and one value per dam/litter was used for statistical analysis.

For all offspring data, one male and one female offspring were randomly selected from each litter. Because offspring originating from the same litter share maternal and perinatal exposures, litter identity was retained in the statistical analyses and male and female offspring from the same litter were treated as matched observations where appropriate. Longitudinal offspring body weights were analyzed using a three-way mixed-effects model (REML), with time, sex, and maternal diet as fixed factors. Time was treated as a repeated measure within individual offspring, and male and female offspring originating from the same litter were treated as matched observations to account for their shared maternal and perinatal exposure. Dam/litter was therefore incorporated as the experimental unit. Because the assumption of sphericity was not imposed, Geisser-Greenhouse correction was applied to effects involving the repeated time factor. The model assessed the main effects of time, sex, and maternal diet and all two- and three-way interactions. Post hoc comparisons between maternal diet groups at individual ages were performed using Šídák’s multiple-comparisons test. =For offspring ipGTT, a three-way repeated measures ANOVA was performed with time, sex, and maternal-diet as factors. For offspring ipGTT, AUC, serum insulin and leptin a two-way ANOVA was performed with maternal diet and sex as factors. Model residual plots for the longitudinal mixed-effects analysis were visually inspected to assess model assumptions and identify potential influential observations. No gross departures from model assumptions or systematic patterns were observed. No observations were excluded from the analyses as statistical outliers.

## Results

### Maternal metabolic outcomes following ketogenic diet consumption before and during pregnancy

At GD 16.5, keto dams exhibited impaired glucose tolerance, as indicated by significantly elevated blood glucose levels at 30 (p = 0.0348) minutes post glucose injection and increased area under the curve (AUC) (p = 0.0059) compared to control dams (Fig 2A and B). Keto dams also weighed significantly more (p = 0.0430) than control dams at this timepoint (Fig 2C).

**Fig 2:**
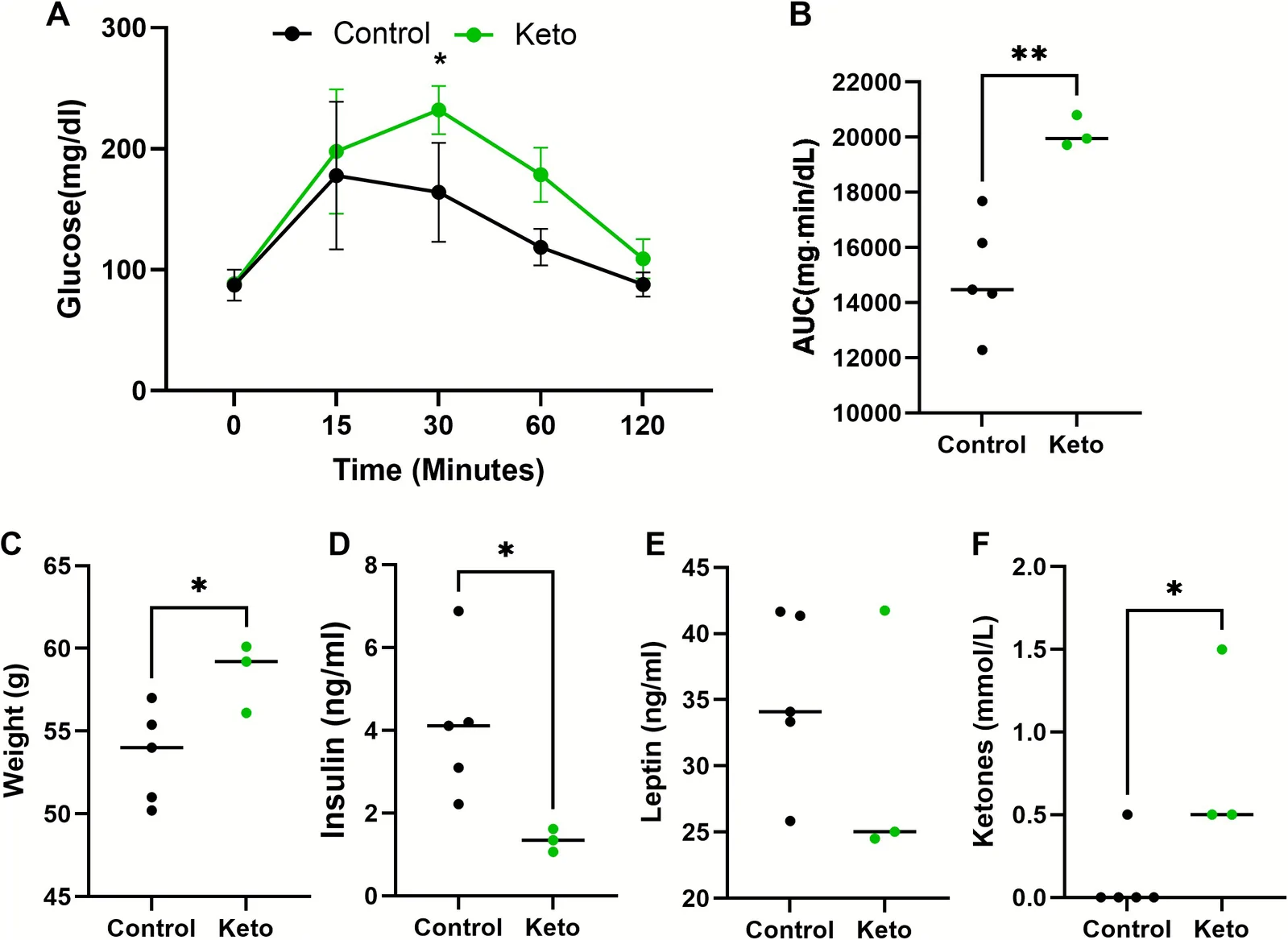
Maternal metabolic outcomes in control and ketogenic diet-fed dams. (A) Blood glucose levels during intraperitoneal glucose tolerance test (IPGTT) at GD 16.5. Blood glucose was significantly increased in keto dams at 30 minutes post-glucose injection (p = 0.0348). (B) Area under the curve (AUC) for IPGTT was significantly increased in keto dams (p = 0.0059). (C) Maternal body weight at GD 16.5 was significantly increased in keto dams (p = 0.0430). (D–E) Serum leptin and insulin levels measured at GD 17.5. Serum leptin did not differ between groups, whereas serum insulin was significantly decreased in keto dams (p = 0.0396). (F) Urinary ketone levels assessed at GD 17.5 were significantly elevated in keto dams (p = 0.0384). Data are presented as mean ± SE. Statistical analysis was performed using two-way repeated measures ANOVA for IPGTT and unpaired Student’s t-tests for other comparisons. Each data point represents one dam (Control n = 5, Keto n = 3). Asterisks displayed in the figure denote statistical significance. *p < 0.05, **p < 0.01.

At GD 17.5 keto dams displayed increased adiposity by gross observation, although serum leptin levels were not different between groups (Fig 2D). Serum insulin levels were significantly decreased (p = 0.0396) in keto dams compared to controls (Fig 2E). Urinary ketone levels were significantly elevated (p = 0.0384) in keto dams, confirming a state of ketosis (Fig 2F).

Mean litter size did not differ between control and keto dams at GD 17.5 (Fig 3A). However, fetuses from keto dams had significantly lower (p = 0.0200) mean fetal weights despite similar litter sizes (Fig 3B), while placental weights and placental efficiency were not different (Fig 3C and D). Together, these exploratory findings identify maternal metabolic alterations and reduced fetal weight in ketogenic diet-fed pregnancies; however, the limited number of independent pregnancies requires cautious interpretation of these observations.

**Fig 3:**
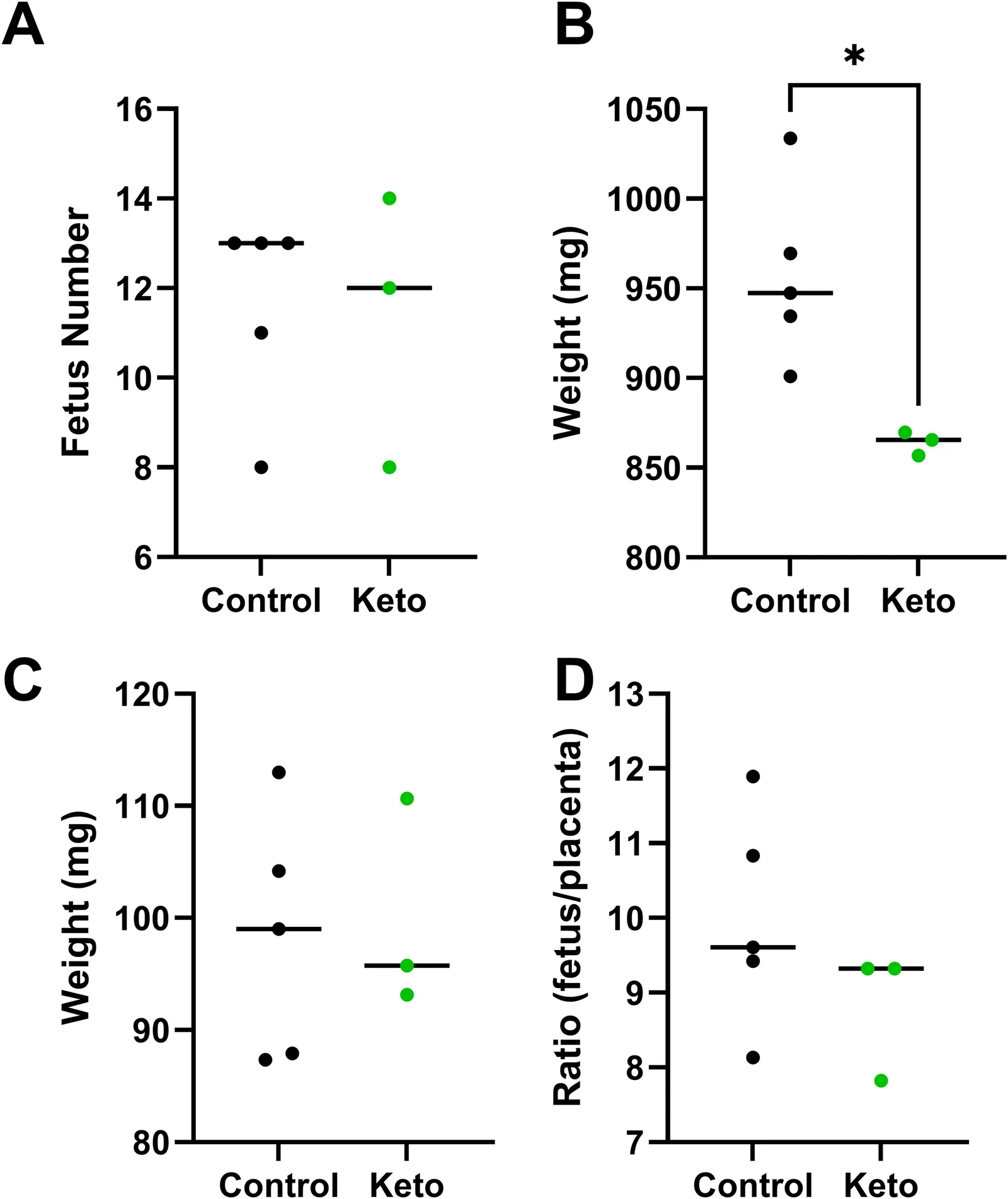
Fetal and placental outcomes at GD 17.5. (A) Litter size per dam. (B) Mean fetal weight per dam was significantly lower in keto pregnancies compared to controls (p = 0.0200). (C) Mean placental weight per dam. (D) Placental efficiency (fetal weight/placental weight). Litter size, placental weight, and placental efficiency did not differ significantly between groups. Each data point represents the mean value per dam. Data are presented as mean ± SE. Statistical analysis was performed using Student’s t-test (Control n = 5, Keto n = 3). Asterisks displayed in the figure denote statistical significance. *p < 0.05.

### Offspring growth and metabolic outcomes following maternal ketogenic diet exposure

Litter size at birth was not different between control and keto fed-dams (10.4 ± 0.7, 10.9 ± 0.7; p = 0.6777). Male and female offspring were weighed weekly from weaning through 12 weeks of age. Longitudinal body weight was analyzed using a three-way mixed-effects model accounting for repeated measures over time and matching male and female offspring within litter. Body weight increased significantly with age (time: F(1.486, 20.81)=523.2, p < 0.0001), and male offspring weighed significantly more than females overall (sex: F(1,14)=32.54, p < 0.0001). There was no significant overall effect of maternal diet on offspring body weight (F (1,126)=2.268, p = 0.1346) and no significant maternal diet x time interaction (F(1.486,20.81)=2.494, p = 0.1186), indicating that postnatal growth trajectories did not significantly differ between control and keto-exposed offspring. A significant time x sex interaction was observed (F(2.226,31.16)=10.14, p = 0.0003), whereas diet x sex (p = 0.4592) and time x diet x sex (p = 0.4934) interactions were not significant (Fig 4). Although keto-exposed offspring tended to have lower body weights at several postnatal time points, considerable overlap was observed between groups, and the longitudinal model did not identify a significant overall maternal diet effect or diet x time interaction. Post hoc comparisons did not identify significant differences between control and keto offspring at individual ages after Šídák correction for multiple comparisons.

**Fig 4:**
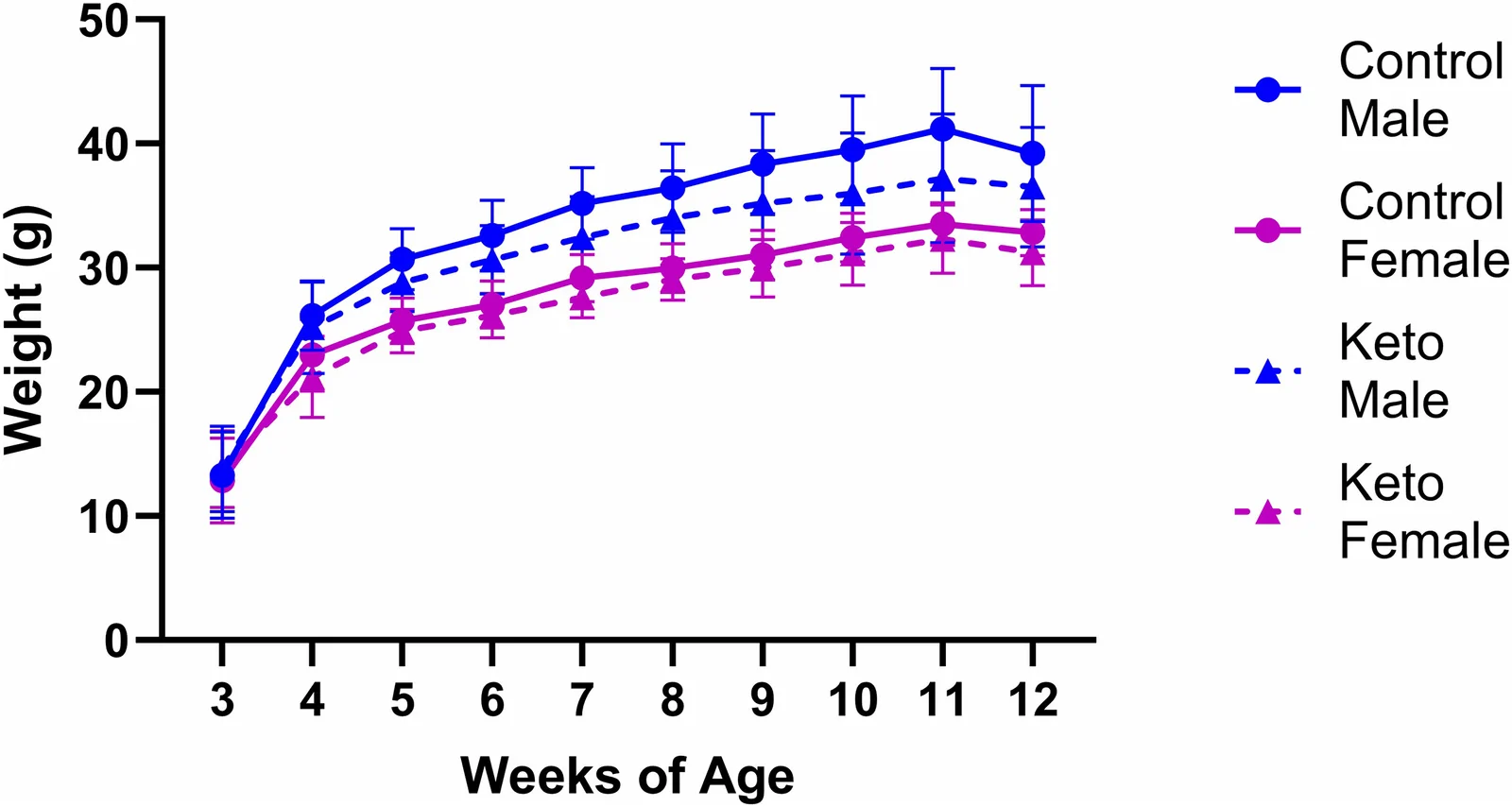
Longitudinal body weight of offspring from control and keto-fed dams. Body weights of one male and one female offspring randomly selected from each litter were measured weekly from weaning through 12 weeks of age (Control n = 9 litters; Keto n = 7 litters). Data are presented as mean ± SE. Longitudinal data were analyzed using a three-way mixed-effects model (REML) with time, sex, and maternal diet as fixed factors; time was treated as a repeated measure and male and female offspring from the same litter were treated as matched observations. Geisser–Greenhouse correction was applied to effects involving time. There were significant effects of time (p < 0.0001) and sex (p < 0.0001), and a significant time × sex interaction (p = 0.0003). The main effect of maternal diet (p = 0.1346), diet × time interaction (p = 0.1186), diet × sex interaction (p = 0.4592), and time × diet × sex interaction (p = 0.4934) were not significant. Post hoc comparisons did not identify significant differences between maternal diet groups at individual ages following ^Šídák^ correction for multiple comparisons.

At 12 weeks of age, glucose tolerance tests revealed that male and female offspring from keto dams exhibited modest but statistically significant improvement in glucose tolerance compared to control male (p = 0.0189) and female (p = 0.0015) offspring (Fig 5B).

**Fig 5:**
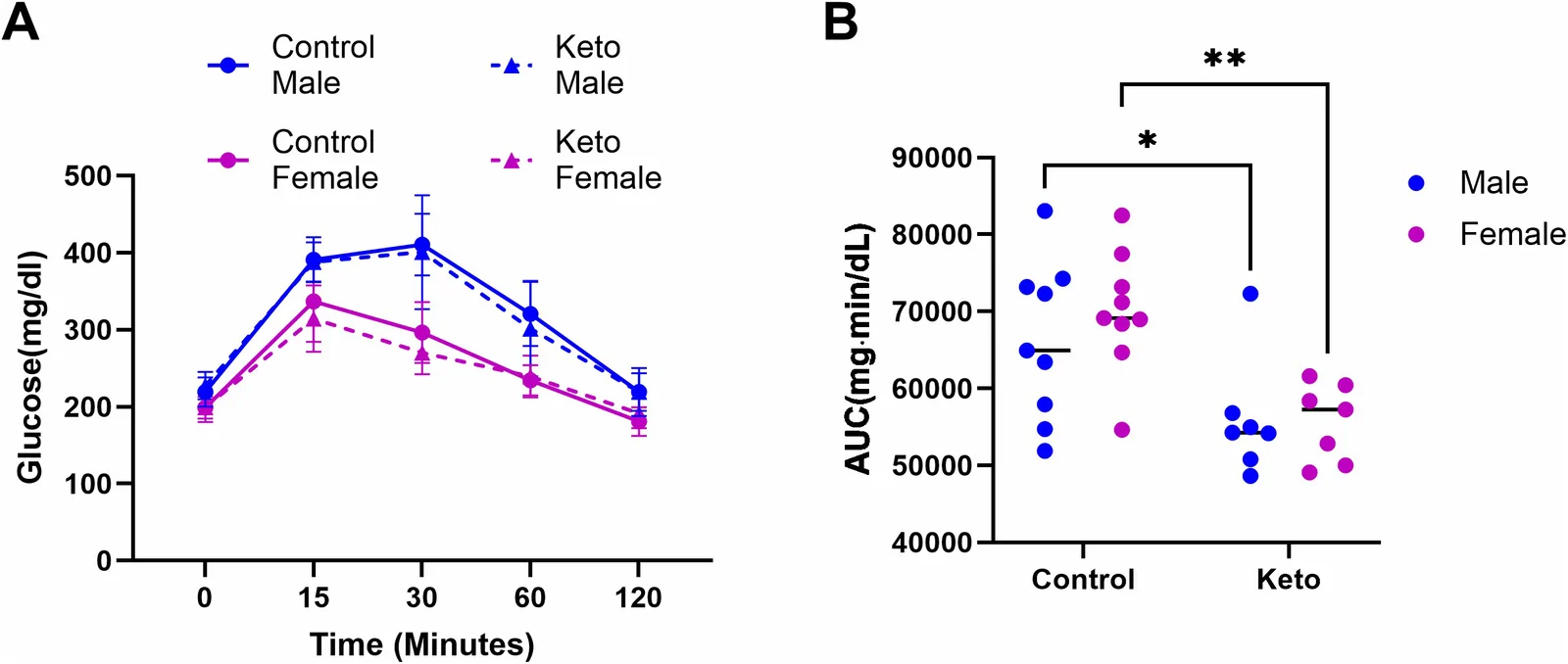
Glucose tolerance in offspring at 12 weeks of age. (A) Blood glucose levels during IPGTT. (B) Area under the curve (AUC). Keto-exposed offspring exhibited significantly lower ipGTT AUC compared with control offspring in both males (p = −0.0189) and females (p = 0.0015). Data represent mean ± SE, with one male and one female per dam included in analysis (Control n = 9 dams, Keto n = 7 dams). Statistical analysis was performed using three-way repeated-measures ANOVA with sex, maternal diet, and time as factors for ipGTT and two-way ANOVA with sex and maternal diet as factors for AUC. Asterisks displayed in the figure denote statistical significance: *p < 0.05, **p < 0.01.

Serum insulin levels were significantly reduced (p = 0.0044) in male keto offspring compared to male controls (Fig 6A), while no differences were observed between female control and keto offspring. Within the control group, male offspring exhibited higher insulin levels than females (p = 0.0029); however, this sex difference was not observed in keto offspring.

**Fig 6:**
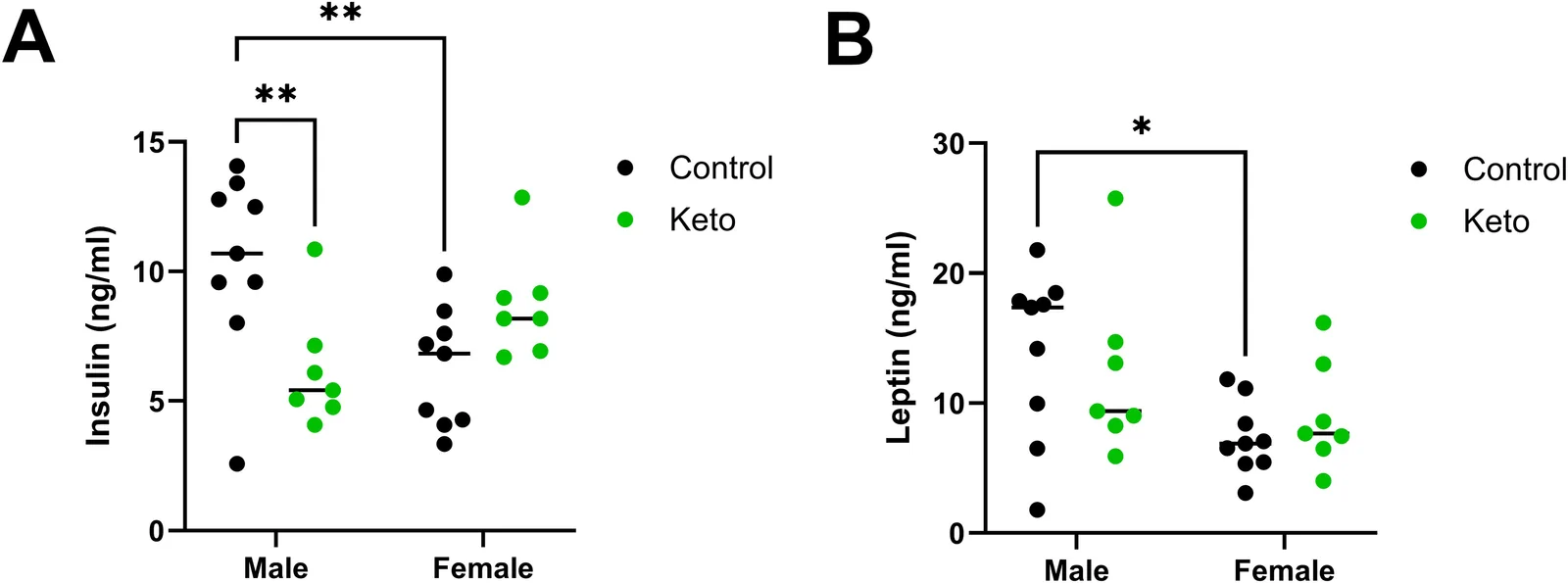
Serum insulin and leptin levels in offspring at 12 weeks of age. (A) Serum insulin concentrations. Serum insulin was significantly lower in male keto offspring compared with male control offspring (p = 0.0044). Within control group, serum insulin was significantly higher in males than females (p = 0.0029). No significant difference between maternal diet groups was observed in female offspring. (B) Serum leptin concentrations. Serum leptin did not differ significantly between maternal diet groups. Within control group, serum leptin was significantly higher in males than females (p = 0.0121), whereas no significant sex difference was observed in keto offspring. Data represent mean ± SE, with one male and one female per dam used for statistical analysis (Control n = 9 dams, Keto n = 7 dams). Statistical analysis was performed using two-way ANOVA with sex and maternal diet as factors. Asterisks displayed in the figure denote statistical significance *p < 0.05, **p < 0.01.

Serum leptin levels did not differ between control and keto offspring (Fig 6B). In control offspring, males exhibited higher leptin levels than females (p = 0.0121), whereas no sex differences were observed in keto offspring.

## Discussion

The findings of this study indicate that maternal consumption of a ketogenic diet was associated with altered maternal glucose homeostasis during pregnancy and reduced fetal growth, as well as with modest alterations in offspring metabolic outcomes. Despite the limited maternal sample size, differences were observed across several measures of maternal physiology, including glucose tolerance, urinary ketones, circulating insulin concentrations, maternal body weight, and fetal weight. While these observations are internally consistent with altered maternal glucose homeostasis and ketosis, they should be considered preliminary given the limited number of independent pregnancies. Notably, key diagnostic criteria for diabetic ketoacidosis, including blood pH [31,32], were not assessed, and therefore a definitive diagnosis of ketoacidosis cannot be made.

Data on the safety and metabolic consequences of ketogenic diet consumption during human pregnancy remain limited. A recent survey of healthcare professionals found that most providers do not recommend ketogenic diets during pregnancy, citing insufficient evidence regarding maternal and fetal safety [33]. In non-pregnant women, particularly those with PCOS, ketogenic diets are often associated with improved metabolic health and weight loss [17,18]. In contrast, the ketogenic diet increased adiposity in our mouse model. This difference likely reflects species-specific metabolic adaptations and differences in diet composition. The ketogenic diet used in this study consisted of 75% fat derived from soybean oil, lard and cocoa butter and is comparable to diets used in prior rodent studies [21–23,25]. Although murine models cannot fully replicate human metabolic response to ketogenic diets, they remain an important preclinical model for investigating the effects of maternal nutritional ketosis and carbohydrate restriction on pregnancy and offspring development under controlled experimental conditions. Consistent with our findings, Sussman et al reported gestational and lactational ketoacidosis leading to maternal mortality in mice [21], although no maternal deaths were observed in the present study, potentially due to differences in mouse strain or experimental conditions.

An alternative interpretation of the metabolic changes observed in the keto dams is that ketogenic diet consumption may augment the normal metabolic adaptations of pregnancy. Pregnancy is characterized by progressive insulin resistance and reduced peripheral glucose utilization, adaptations that promote glucose availability for the developing fetus [34,35]. Under conditions of severe carbohydrate restriction, these physiological adaptations may be further exaggerated, resulting in altered glucose homeostasis rather than outright metabolic dysfunction. Additional studies assessing insulin sensitivity, circulating lipids, blood ketone concentrations, and whole body energy metabolism will be required to distinguish adaptive metabolic responses from pathologic metabolic impairment.

Although maternal metabolic alterations were observed, offspring exhibited relatively modest metabolic alterations during early adulthood. Offspring exposed to a maternal ketogenic diet tended to have lower body weights during early postnatal development, although the longitudinal mixed-effects analysis identified neither a significant overall effect of maternal diet nor a significant diet x time interaction. Thus, the present data do not provide statistical evidence for a distinct postnatal growth trajectory in keto-exposed offspring. Body weights converged visually between groups by 12 weeks of age but this pattern should not be interpreted as definitive evidence of catch-up growth.

Interestingly, offspring from keto dams exhibited a modest improvement in glucose tolerance at 12 weeks of age. Although statistically significant, the magnitude of this effect was small, and its physiological relevance remains unclear. This finding suggests that early metabolic adaptations may occur in response to maternal ketogenic diet exposure, but do not necessarily indicate improved long-term metabolic health.

Sex-specific effects were also observed. In control offspring, males exhibited higher circulating insulin and leptin levels than females, consistent with known sex differences in metabolic regulation [28,36,37]. In contrast, these sex differences were attenuated or absent in offspring exposed to the ketogenic diet, suggesting that maternal diet may influence the development of sexually dimorphic metabolic pathways.

The reduced fetal weight observed in keto-exposed pregnancies despite comparable litter sizes suggests impaired fetal growth in response to maternal ketogenic diet consumption. However, because these findings are based on a limited number of pregnancies, confirmation in larger cohorts will be necessary. Reduced fetal size may reflect alterations in the intrauterine environment associated with maternal ketosis and impaired glucose metabolism. Ketone bodies are known to cross the placenta barrier and may also be present in breast milk, potentially exposing the developing fetus and neonate to altered nutrient availability [31,38]. However, ketone concentrations were not directly measured in fetal or neonatal tissues in this study, and the mechanisms underlying fetal growth restriction remain to be determined.

It is important to note that offspring health cannot be assessed solely by the absence of early metabolic dysfunction. Previous studies have shown that maternal ketogenic exposure can adversely affect offspring neurodevelopment [20,21]. Keto offspring have been reported to exhibit reduced white matter volume, impairing cognitive and motor development, likely due to altered lipid metabolism and elevated prenatal ketone exposure [19]. Other studies demonstrate reduced size of crucial midbrain structures alongside disproportionate enlargement of the hypothalamus and medulla [20], changes that may contribute to long-term neurological and physiological dysfunction.

This study has several limitations. Maternal and offspring outcomes were assessed in separate cohorts, precluding direct correlation analyses between maternal metabolic status and offspring phenotypes. Additionally, the sample size in the maternal cohort was limited, consisting of five control and three keto dams. Although statistically significant differences were observed across several independent maternal outcomes, the small sample size limits statistical power and increases the potential influence of biological variability. Accordingly, the maternal and fetal findings from Experiment 1 should be considered exploratory and hypothesis-generating rather than definitive, and confirmation in larger, independently powered cohorts will be necessary to establish their reproducibility. Furthermore, litters were intentionally left undisturbed immediately after birth to minimize maternal stress and pup cannibalism. Consequently, individual pup body weights, maternal body weights during lactation, neonatal survival, and litter-level sex ratios were not recorded. Therefore, we cannot distinguish prenatal from lactational influences of maternal ketogenic diet exposure. Future studies incorporating larger maternal cohorts, longitudinal assessments, and comprehensive metabolic assessments, including lipid metabolism, oxidative stress, and placental function, will be important to better define the impact of maternal ketogenic diet exposure on offspring health.

In conclusion, this exploratory study identified associations between maternal ketogenic diet consumption, altered maternal glucose homeostasis and reduced fetal growth in this mouse model, while the independent offspring cohort demonstrated modest and sex-specific metabolic changes during early adulthood. Given the limited number of independent pregnancies in Experiment 1, the maternal and fetal findings should be interpreted as preliminary and require confirmation in larger studies. Together, these findings highlight the need for further investigation into the maternal and long-term offspring consequences of ketogenic diet exposure during pregnancy.

## Supporting information

S1 Table Diet Composition Breakdown as provided by Research Diets. (DOCX)

S2 Table Reproductive Characteristics from Experiment 1 and 2. (DOCX)
