# Supplementary material for: Effects of a maternal ketogenic diet on maternal and offspring metabolic health in mice
Source: PLoS One. 2026 Oct 21;21(10):e0356015. doi: 10.1371/journal.pone.0356015 (PMC13686513; doi:10.1371/journal.pone.0356015)
Supplement: Supplementary file 1 [file pone.0356015.s001.docx]

| **Diet** | Ketogenic  (D14090501) | |  | Control  (D12450K) | |
| --- | --- | --- | --- | --- | --- |
|  | gm% | *kcal%* |  | gm% | *kcal%* |
| Protein | 29.5 | *20* |  | 19.2 | *20* |
| Carbohydrate | 7.4 | *5* |  | 67.3 | *70* |
| Fat | 49.1 | 75 |  | 4.3 | 10 |
| Total |  | *100* |  |  | *100* |
| kcal/gm | 5.89 |  |  | 3.85 |  |
|  |  |  |  |  |  |
| **Ingredient** | **gm** | ***kcal*** |  | **gm** | ***kcal*** |
| Casein | 200 | *800* |  | 200 | *800* |
| L-Cystine | 3 | *12* |  | 3 | *12* |
| Corn Starch | 20.5 | *82* |  | 550 | *2200* |
| Maltodextrin 10 | 20.5 | *82* |  | 150 | *600* |
| Sucrose | 0 | *0* |  | 0 | *0* |
| Cellulose | 50 | *0* |  | 50 | *0* |
| Soybean Oil | 25 | *225* |  | 25 | *225* |
| Lard | 113 | *1017* |  | 20 | *180* |
| Cocoa Butter | 200 | *1800* |  | 0 | *0* |
| Mineral Mix S10026 | 10 | *0* |  | 10 | *0* |
| DiCalcium Phosphate | 13 | *0* |  | 13 | *0* |
| Calcium Carbonate | 5.5 | *0* |  | 5.5 | *0* |
| Potassium Citrate, 1 H2O | 16.5 | *0* |  | 16.5 | *0* |
| Vitamin Mix V10001 | 10 | *40* |  | 10 | *40* |
| Choline Bitartrate | 2 | *0* |  | 2 | *0* |
| FD&C Yellow Dye #5 | 0  0.05  0 | *0* |  | 0  0.025  0.025 | *0* |
| FD&C Red Dye #40 |  | *0* |  |  | *0* |
| FD&C Blue Dye #1 |  | *0* |  |  | *0* |
| **Total** | **689.1** | ***4058*** |  | **1055.1** | ***4057*** |
|  |  |  |  |  |  |
| Saturated Fat | 158.91 | 1,430.18 |  | 10.14 | 91.22 |
| Monounsaturated Fat | 110.89 | 997.98 |  | 12.83 | 115.47 |
| Polyunsaturated Fat | 51.09 | 459.77 |  | 20.18 | 181.58 |
|  |  |  |  |  |  |
| Saturated Fat (kcal%) | 35.24 |  |  | 2.25 |  |
| Monounsaturated Fat (kcal%) | 24.59 |  |  | 2.85 |  |
| Polyunsaturated Fat (kcal%) | 11.33 |  |  | 4.48 |  |

Supporting Information: S1 Table. Diet Composition
