# Supplementary material for: Effects of a maternal ketogenic diet on maternal and offspring metabolic health in mice
Source: PLoS One. 2026 Oct 21;21(10):e0356015. doi: 10.1371/journal.pone.0356015 (PMC13686513; doi:10.1371/journal.pone.0356015)
Supplement: Supplementary file 2 [file pone.0356015.s002.docx]

Supporting Information: S2 Table. Reproductive Characteristics

| **Variable** | **Control** | **Keto** | **P value** |
| --- | --- | --- | --- |
| Females Enrolled | 20 | 20 | - |
| Pregnant Dams | 14 | 10 | - |
| Pregnancy Rate (%) | 70 | 50 | 0.2065 |
| Dams in Exp. 1 | 5 | 3 | - |
| - Exp. 1 Litter Size | 11.6±1.0 | 11.3±1.8 | 0.9026 |
| Dams in Exp. 2 | 9 | 7 | - |
| - Exp. 2 Litter Size | 10.4±0.7 | 10.9±0.7 | 0.6777 |
